# Psychological Toll of the COVID-19 Pandemic: An In-Depth Exploration of Anxiety, Depression, and Insomnia and the Influence of Quarantine Measures on Daily Life

**DOI:** 10.3390/healthcare11172418

**Published:** 2023-08-29

**Authors:** Musheer A. Aljaberi, Mohammed A. Al-Sharafi, Md. Uzir Hossain Uzir, Aiche Sabah, Amira Mohammed Ali, Kuo-Hsin Lee, Abdulsamad Alsalahi, Sarah Noman, Chung-Ying Lin

**Affiliations:** 1 Faculty of Medicine and Health Sciences, Taiz University, Taiz 6803, Yemen; 2 Faculty of Nursing and Applied Sciences, Lincoln University College, Petaling Jaya 47301, Malaysia; 3 Department of Community Health, Faculty of Medicine & Health Sciences, Universiti Putra Malaysia, Serdang 43300, Malaysia; saranoman12@gmail.com; 4 Institute of Informatics and Computing in Energy, Universiti Tenaga Nasional, Putrajaya Campus, Kajang 43000, Malaysia; mohamed.a.alsharafi@gmail.com; 5 Faculty of Business and Accountancy, Lincoln University College, Petaling Jaya 47301, Malaysia; hossainuzir@gmail.com; 6 Faculty of Human and Social Sciences, Hassiba Benbouali University of Chlef, Chlef 02076, Algeria; s.aiche@univ-chlef.dz; 7 Department of Psychiatric Nursing and Mental Health, Faculty of Nursing, Alexandria University, Smouha, Alexandria 21527, Egypt; amira.mohali@alexu.edu.eg; 8 Department of Emergency Medicine, E-Da Dachang Hospital, I-Shou University, Kaohsiung City 824, Taiwan; 9 School of Medicine, College of Medicine, I-Shou University, No. 8, Yi-Da Road, Jiao-Su Village, Yan-Chao District, Kaohsiung City 824, Taiwan; 10 Department of Pharmacology, Faculty of Pharmacy, Sana’a University, Sana’a 1247, Yemen; ahmedsamad28@yahoo.com; 11 Institute of Allied Health Sciences, College of Medicine, National Cheng Kung University, Tainan 701, Taiwan; cylin36933@gmail.com

**Keywords:** post-traumatic stress, anxiety, depression, insomnia, quarantine, COVID-19, mental health, well-being, life threats, shortage of healthcare

## Abstract

The COVID-19 pandemic, on a global scale, has prompted multifaceted challenges, including a notable psychological toll on the general population. This study uses mixed-method approach for a nuanced exploration of these experiences. Using a phenomenological strategy, qualitative responses from 999 participants were analyzed regarding their pandemic-induced anxiety and the influence of quarantine measures on their lives. Quantitative measures, including the revised Impact of Event Scale (IES-R), patient health questionnaire-9 (PHQ-9), the seven-item generalized anxiety disorder assessment (GAD-7), and Insomnia Severity Index (ISI), were used to quantify trauma, depression, anxiety, and insomnia attributed to COVID-19. Partial least squares structural equation modeling (PLS-SEM) was utilized for quantitative data analysis. The anxiety-related responses were mainly clustered into four themes: life threats, support shortage, economic consequences, and disruptions to family and social life. Subthemes that addressed the perceived effects encapsulated disruptions to academic and professional lives, familial and social relationships, psychopathological stress, and movement limitations. The findings from quantitative analysis revealed the significant associations between COVID-19-related trauma and symptoms of anxiety, depression, and insomnia, as indicated by coefficients exceeding 0.10 (all *z*-values > 1.96; *p*-values < 0.05). In conclusion, the findings underscore COVID-19’s role in escalating anxiety, influenced by various factors, and its disruptive effects on daily life due to quarantine measures. The strong associations between the pandemic and the symptoms of depression, anxiety, and insomnia underscore the urgency of comprehensive psychological and public health interventions to alleviate these impacts.

## 1. Introduction

Breaking out in late 2019, the new Coronavirus Disease 2019 (COVID-19) has produced multiple waves, leading to significant global health impacts [1,2]. On 16 January 2023, the World Health Organization (WHO) reported over 662 million confirmed cases, with more than 6.7 million of these ending in fatalities worldwide [3,4,5]. The pandemic has resulted in considerable economic damages and financial distress due to extended lockdowns and business closures, with varying effects on diverse demographic groups [6,7,8]; it also profoundly impacted various aspects of human life (mental and physical health, education, social interactions, and healthcare delivery) [5,9,10,11,12,13].

Of physical and psychological behaviors, this pandemic significantly affected the mental behaviors of individuals, social groups, and organizations, including emotions, cognition, behavior, overall mental health, and related psychosocial factors [14]. The COVID-19 pandemic and associated quarantine and lockdowns have sparked various psychological and behavioral responses, including depression, anxiety, stress, sleep disorders, increased demand for healthcare, and even suicidal ideation [5,15,16,17,18], with a remarkable overall increase in the monthly suicide rate [19,20,21]. These widespread effects could dramatically affect general mental well-being in a continuous spectrum [22]. These findings indicate that pandemics negatively affect mental health outcomes, and that mental health support is necessary to mitigate the negative psychological impact of pandemics. Various factors, such as COVID-19-related misinformation, shortage of treatments, inequity in vaccine distribution, and disease-associated stigma, have been linked to significant impacts on mental health [23,24,25,26]. COVID-19-induced traumatic emotions, in turn, have been associated with post-traumatic stress disorder (PTSD), which is associated with increased mental symptoms and maladaptive behaviors [4,5,24].

Pre-existing mental or physical illnesses have been identified among the key risk factors for psychological disorders during pandemics [27,28]. Evidence suggests worse mental health outcomes during the pandemic among women and young people (aged 18–29 years), particularly those from socially disadvantaged backgrounds [29]. Furthermore, certain demographic groups, such as older adults, children, and racial and ethnic minorities, are also differentially affected in this critical time of history [7,8]. Research consistently draws on rigorous data analysis and relevant literature to comprehensively understand the psychological impacts of COVID-19 quarantine. Given the dynamic, complex, and extensive nature of the COVID-19 pandemic-related influence [30], mixed-method approach, which involves collecting and analyzing qualitative and quantitative data, would offer a more comprehensive understanding of the psychological experiences and the associated mental health outcomes amidst the COVID-19 pandemic [31]. Accordingly, this study investigates the psychological experiences related to the pandemic and quarantine’s effects on life. It quantifies the association of these experiences with mental symptoms of anxiety, depression, post-traumatic stress, and insomnia. The current study is expected to contribute to the literature on psychological research, mental-medical literature, and depression-anxiety-stress theory. Now that the pandemic may be ending, future pandemics are still possible, and the current study results provide policymakers and healthcare professionals with guidance in approaching the challenges of long mental effects of COVID-19. They may also provide insights for addressing similar future pandemics.

## 2. Hypotheses Development

The existing literature provides a solid foundation for the hypothesis’s development related to the psychological trauma caused by the COVID-19 pandemic. The pandemic’s wide-reaching impacts on mental health have been widely recognized, with anxiety, depression, and insomnia frequently cited as notable symptoms [15,16,17,32,33,34,35,36,37,38,39,40,41,42]. The profound psychological and emotional stresses associated with the pandemic have spurred the development of the following hypotheses:

### 2.1. Anxiety

Anxiety has long been established as a common response to stressful or traumatic events [40,43]. Multiple studies have highlighted the significance of examining anxiety in the context of the COVID-19 pandemic. The general population has witnessed a surge in anxiety, fear, and stress as a direct consequence of the pandemic. Researchers have identified specific stressors related to COVID-19 that contribute significantly to these heightened anxiety levels [27,44,45,46]. The impact of the pandemic on anxiety has been far-reaching, leading individuals to experience hopelessness, sadness, and a perceived lack of control, which, in turn, have manifested in undesirable societal behaviors [47]. Frontline medical professionals engaged in the battle against COVID-19 have been particularly susceptible to elevated anxiety levels [48,49,50]. Vulnerable social groups, including individuals with pre-existing emotional disorders, young adults, the unemployed, singles, those with limited education, and women, require additional support to address the stress and anxiety associated with the pandemic [51]. In India, anxiety levels among the population have reached alarmingly high rates, with over 80% of participants in one study expressing a need for mental health care [44]. Surgeons in Nigeria have also reported anxiety stemming from concerns about their loved ones contracting the virus [52].

Furthermore, research has established a significant correlation between anxiety and COVID-19-related trauma [53,54,55,56]. The COVID-19 pandemic has triggered elevated anxiety levels in the general population due to the uncertainty and fear surrounding the virus. Moreover, the significant disruptions to daily life and societal structures have intensified these anxiety levels. Maladaptive coping mechanisms have been identified as partially mediating the relationship between intolerance of uncertainty and psychological distress [57]. Conversely, perceived social support has shown a noteworthy impact in reducing anxiety levels [58]. A meta-analysis of community studies has revealed that the estimated prevalence of anxiety during the pandemic is three times higher than the typical prevalence of anxiety disorders, reaching 25% [59]. Intolerance of uncertainty regarding the COVID-19 pandemic has particularly affected anxiety and depressive symptoms during quarantine, especially among young women, who are more intolerant of uncertainty [60]. Considering these findings, it is evident that anxiety levels have significantly increased during the pandemic. However, further research is needed to fully understand the intricate association between anxiety and various aspects of the pandemic [61]. A continued investigation will contribute to a better comprehension of the impact of anxiety and aid in developing targeted interventions to address the mental health challenges posed by the COVID-19 pandemic. Consequently, the first hypothesis posits:

**H1:**  *Anxiety is significantly associated with COVID-19-related psychological trauma.*

### 2.2. Depression

Depressive symptoms related to the COVID-19 pandemic have been extensively reported, stemming from various factors such as isolation, loss of employment, health concerns, and bereavement [32,41]. Research suggests that COVID-19 significantly impacts depression, particularly among adolescents who face challenges such as limited social contact, lack of space for activities, and uncertainty about the future [62]. Additionally, healthcare professionals working in COVID-19 hospitals have been found to experience unusually high levels of depression [63], and a substantial proportion of hospitalized patients with COVID-19 exhibit symptoms of depression, with a reported prevalence of 75% [64]. Depression is also commonly observed in patients with mild to moderate COVID-19 disease [65], and symptoms of anxiety and depression frequently manifest themselves as psychological responses to the pandemic, potentially linked to disturbed sleep [66]. A meta-analysis indicates a depression prevalence of 27.60% [67], and a systematic review demonstrates higher depression scores in the general population compared to pre-COVID-19 levels [68]. The review also reports a combined prevalence of all forms of depression of 20% among a study population of 113,285 individuals [69].

Additionally, there has been a slight increase in depression levels during the pandemic [70]. Previous research suggests a link between depressive symptoms and the emergence of psychological trauma in both COVID-19 survivors and healthcare professionals. Depressive symptoms have been inversely correlated with gray matter volume in the anterior cingulate and insular cortex, previously associated with depression and post-traumatic stress disorder [71]. Healthcare professionals who work with COVID-19 patients are at increased risk of experiencing various mental disorders, including depression, anxiety, distress, insomnia, and vicarious trauma [72]. These studies highlight the importance of addressing the impact of the pandemic on depression, as it has caused significant emotional distress and profoundly influenced mental well-being.

Furthermore, these studies indicate that depressive symptoms are frequently reported in connection with the COVID-19 pandemic. In addition, symptoms of depression can contribute to the development of psychological trauma among survivors of COVID-19 and healthcare professionals. Therefore, the second hypothesis is proposed as follows.

**H2:**  *Depression is significantly associated with COVID-19-related psychological trauma.*

### 2.3. Insomnia

Sleep disturbances, including insomnia, are commonly observed in traumatic stress responses [42]. The stress and anxiety provoked by the pandemic may disrupt normal sleep patterns, leading to insomnia. This sleep disorder, in turn, might exacerbate the psychological trauma related to COVID-19. Numerous studies have indicated that COVID-19 and related factors may influence insomnia [10]. Insomnia can be caused by COVID-19 infection itself, resulting from hypoxia and systemic inflammatory mediators [73]. Depression has also emerged as a significant predictor of insomnia during the pandemic [74]. In China, insomnia symptoms were observed in more than a third of the population in the early and late stages of the pandemic [75]. A study conducted in France found that COVID-19-related worries and feelings of loneliness were the main contributing factors to clinical insomnia [76].

Furthermore, studies suggest a strong association between the psychological trauma of COVID-19 and a high prevalence of insomnia. Insomnia was more severe in women, young individuals, those residing in the epicenters of COVID-19, and those with a high degree of threat from the virus [77]. COVID-19 survivors and healthcare workers also had a high rate of insomnia [78,79]. COVID-19 has been associated with a specific spectrum of sleep changes known as COVID-somnia [80]. Another study reported a prevalence of 42.8% for insomnia disorder among COVID-19 patients in Wuhan, China [81]. Sleep problems were identified in 37.6% of the Greek population surveyed during the pandemic [82]. In Indonesia, nearly half of the COVID-19 patients isolated in healthcare facilities were found to suffer from insomnia [83]. These findings underscore the impact of COVID-19 on sleep patterns and emphasize the need for increased attention and support for people suffering from insomnia during the pandemic. Therefore, the third hypothesis is:

**H3:**  *Insomnia is significantly associated with COVID-19-related psychological trauma.*

## 3. Materials and Methods

### 3.1. Study Subjects

In this cross-sectional study, convenience sampling was employed through an online survey to gauge the public’s immediate psychological responses during the COVID-19 pandemic. Due to lockdown measures, participants were reached via various online platforms. The survey, constructed on Google Forms, was disseminated through a hyperlink. From March to July 2020, 1020 participants from 20 distinct countries were recruited using purposive sampling. Of these, 21 participants did not complete all the survey items, so the final analysis included 999 participants. The demographic details of these participants are presented in Supplementary Table S1.

### 3.2. Research Design

A convergent mixed-method design was utilized in this study, allowing for the simultaneous collection and initial separate analysis of qualitative and quantitative data, followed by a combined analysis in the subsequent stage [84,85,86]. This methodological approach facilitates data triangulation, with both qualitative and quantitative findings mutually supporting each other. Quantitative data was procured through participant responses to four distinct scales. Firstly, the Impact of Event Scale-Revised (IES-R) was used, which consists of 22 items categorized into three subscales: intrusion, avoidance, and hyperarousal [87]. The IES-R is a validated self-administered questionnaire applicable across different populations, capturing the primary characteristics of PTSD relevant to a particular trauma, in this case, COVID-19 [88]. The second measure used was the patient health questionnaire-9 (PHQ), a nine-item self-report tool for depression. The PHQ was first developed using the diagnostic criteria of the Diagnostic and Statistical Manual of Mental Disorders, fourth edition (DSM-IV). Moreover, the PHQ has been found to be comparable to the latest version of DSM (i.e., DSM-5) with widespread use in the research [89,90,91,92,93,94,95,96]. This scale was used to assess the severity of depressive symptoms over the past two weeks in greater depth.

The third instrument was the seven-item Generalized Anxiety Disorder scale (GAD), which assessed anxiety symptom severity over the previous two weeks [97]. Lastly, the study utilized the Insomnia Severity Index (ISI), a seven-item self-report index for assessing the severity of different stages of insomnia: initial, middle, and late [98]. As discussed in the subsequent results section, the reliability and validity of these scales affirm their psychometric adequacy for this study.

### 3.3. Statistical Analysis

The study was conducted using a descriptive and correlational design grounded in covariance matrix analysis. Qualitative data were collected through two open-ended questions included in the research survey:

***-Do you feel anxious about the spread of coronavirus? If yes, What makes you anxious about the spread of COVID-19?***

***-Has the COVID-19 quarantine affected your life? If yes, please specify?***

Colaizzi’s phenomenological analysis, a widely recognized descriptive method in psychology, was utilized to delve into the essence of the phenomena under investigation [99,100]. This method systematically analyses phenomenological data to isolate the critical elements pertinent to the description of the phenomenon.

The participants’ responses to the open-ended questions were analyzed following Colaizzi’s method, using Excel and the Statistical Package for the Social Sciences (SPSS-25) to code and categorize responses into thematic clusters [100,101,102]. This methodical process involved several steps: repeated and thorough reading of the responses; extraction of significant ideas and statements about COVID-19 experiences; formulation of meanings based on these statements; and categorization of formulated meanings into theme clusters, culminating in an exhaustive description of COVID-19-related experiences. This process was initially conducted by one author, then reviewed and refined by two additional authors, thereby ensuring the reliability of the qualitative findings [103].

Simultaneously, the study employed a quantitative approach to examine the influence of GAD, PHQ, and ISI scores on the IES-R scores. Hypotheses were proposed suggesting significant associations between GAD, PHQ, ISI, and IES-R in the context of COVID-19. The invariance of measurements was tested across different countries using SPSS ANOVA. All observed differences were statistically significant when the *p*-value was <0.05 at a 95% confidence level. Effect sizes were calculated using eta square (η^2^ = sum of squares in group effects/total sum of squares in the ANOVA). The magnitude of eta squared is explained according to Cohen’s suggestion: eta squared 0.01 as a small effect, 0.06 as a moderate effect, and 0.14 as a large effect [104,105,106,107].

Partial least square structural equation modeling (PLS-SEM) was performed using SmartPLS software to test these hypotheses. In this study, PLS-SEM was chosen over traditional Covariance-Based Structural Equation Modeling (CB-SEM) for several reasons that align with our research’s specific characteristics and objectives [108,109]. PLS-SEM was chosen as the statistical tool for its suitability for analyzing continuous and categorical variables, particularly in this study’s exploratory nature, and limited theoretical foundation [108,110,111,112,113]. The study’s exploratory approach required a statistical method that could accommodate complex relationships and multiple constructs without relying on rigid theoretical assumptions. Moreover, the small sample size necessitated an analysis that is robust to non-normal data and outliers [114,115]. The modeling process involved two primary stages: first, evaluating the reliability and validity of the study measures through the assessment of the measurement model, and second, hypothesis testing by assessing the structural model. To ensure robust and reliable results and to obtain accurate estimates of model parameters, bootstrapping with 5000 random samples was performed. The use of 5000 bootstrap samples allowed to obtain more stable parameter estimates, precise confidence intervals, and reliable *p*-values for hypothesis testing [116,117] at 95% confidence interval with bias-corrected approach. Numerous studies across various fields have widely employed PLS-SEM due to its versatility and advantages [118,119,120,121,122]. These methodological choices in PLS-SEM, along with the use of bootstrapping, provided a comprehensive and rigorous exploration of the psychological toll of the COVID-19 pandemic. The findings offer valuable insights into the associations between various psychological factors and COVID-19-related trauma, contributing to the growing understanding of the pandemic’s impact on mental health.

## 4. Results

### 4.1. Qualitative Results

#### 4.1.1. Q1: What Makes You Anxious about the Spread of COVID-19?

The participants were initially asked, “Do you feel anxious about the spread of coronavirus? If yes, What makes you anxious about the spread of COVID-19?” Their responses were grouped into several subthemes, including fear of death (increasing number of deaths), life-threatening disease, high infectivity, shortage of vaccines/treatment, inadequate healthcare availability, lockdown and quarantine, economic shutdown, unemployment, worries about family, and disrupted social life. Notably, a sizable portion of the sample (313; 31.3%) indicated they did not feel anxious about COVID-19’s spread. Upon further thematic analysis, these subthemes were organized into four overarching themes (Table 1):

a. Life treats (death, life-threatening disease, highly infectious);
b. Shortage of support (shortage/unavailability of vaccines, treatment, and inadequate healthcare);
c. Economic impact (lockdown and quarantine, economic shutdown, unemployment);
d. Family and social life (worries about family, disrupted social life).

These main themes (Life threats, Shortage of support, Economic impact, and Family and social life) accounted for 42.44%, 8.11%, 13.01%, and 5.11% of the responses, respectively. The main themes and subthemes are depicted in Table 1. According to the data, 686 participants (68.67%) reported feeling anxious about the spread of the coronavirus, providing reasons for their concerns.

In response to this question, we found responses from the whole sample used in the study (*n* = 999). We analyzed their responses based on procedures discussed in the above materials and methods and deduced several themes for answering the above question (Table 1).

First Theme: Life Threats

The first main theme consisted of subthemes such as death (increasing number of deaths), life-threatening, and highly infectious diseases. The subtheme of death refers to the increasing number of deaths due to the spread of COVID-19. Participants used several vocabularies and words to express this theme, such as “people’s death”, “its deadly nature”, “the high death rate”, “the number of deaths is increasing around the globe”, “very deadly virus”, and “many people died.” that was reported by 123 participants (12.7%) from the totality of the sample (999). On the other hand, 32 (3.20%) of the participants reported COVID-19 as a life-threatening disease that caused death or reason for death. Another subtheme of the life threat’s theme is the infectious nature of COVID-19, which was reported by 269 (26.93%). People think this disease can transmit from one person to another anytime by anyone with different methods of transmission, which cause them anxiety.

Second Theme: Shortage of support

The participants expressed that the spread of COVID-19 was a source of anxiety as there are no vaccines, no cure for the virus, no effective treatments, etc. During the COVID-19 spread, a vaccine was not invented; all healthcare services, such as hospitals, clinics, and community centers, were overloaded with patients. There were 81 participants (8.10%) who expressed thoughts in line with this theme and thought insufficient support and the health care services.

Third Theme: Economic Impact

Study participants reported anxiety over the spread of COVID-19 resulting from the economic downturn. They expressed results such as “*economic downturn, it cripples the economy and life in general, economic impact, economic stability, economic recession, the economy is collapsing, the economy is down, the economic situation may affect future hopes of getting a good job and income*”. Participants expressed that the economic downturn had negative consequences, such as job and financial crises. They reported that the spreading of COVID-19 was the cause of the lockdown and quarantine. They expressed concerns such as “*lockdown, I fear lockdown in my city, it is stressful, lockdown seems depressing, restricted movements, we don’t want social distancing, the continuation of lockdown, quarantine, it’s difficult to be under quarantine, quarantine restricts fieldwork*”, etc. In addition, participants stated that lockdown had negative consequences, such as psychopathological outcomes (e.g., depression, anxiety, stress, and working from home). Joblessness was obvious, and many settled employees were sacked from their organizations. New jobseekers failed to find jobs.

Fourth Theme: Family and Social Life

Under this theoretical theme called “family”, participants expressed their worries about family members affected by COVID-19. Subjects expressed reactions such as “*I worry about my family, I am concerned should anyone from my family get infected, especially the elders*, *I cannot stop thinking about my family*”. There were 53 participants in this theme (5.3%) from the totality of samples (999). Different subject themes were coded by participants’ responses that described more than one issue mentioned above. These themes included the participants who reported that COVID-19 spreading changed their lifestyles wholly and radically, affecting numerous aspects of their lives. In brief, this theme included either participants who reported more than the previously mentioned themes or who reported that the spreading of COVID-19 changed their whole life, with reasons such as “*stop the life work, acute pain and dyspnea, media, fake news, careless people, social media and newspapers, life difficulty, expenses, life paralyses*, *community awareness, and the future seems black*”.

#### 4.1.2. Q2: Has the COVID-19 Quarantine Affected Your Life? If Yes, Please Specify?

The second question investigated the effect of quarantine on life. The answers provided by the respondents were sorted and categorized into themes (Table 2) such as academic and schooling life, family life and friendship interruption, job/work and business interruption, mixed issues, psychopathological pressure, movement restrictions, idly staying at home, income loss or no income, time management, and no travel or tour. The most prominent negative effects of quarantine that were reported are academic and schooling life and psychological pressure, totaling 19% and 10%, respectively. Some miscellaneous impacts were found in this study.

As Table 2 shows, the sub-themes generated from the answer to the second question of the effect of the quarantine due to COVID-19 on regular life were as follows;

Subtheme (1): Academic and Schooling Life Interruption

Under this “Academic and Schooling Life” categorization, participants used several vocabularies and words with the same meaning and ideas for academic life, such as no study, lab work, classes, lectures, research, education, or educational activities. The participants reflected on their experiences as undergraduate students, higher studies researchers, and university staff lecturers. They reported that COVID-19 “*halted research’s progress and affected their education as they could not continue their research. Our lab work has been stopped, and we are unable to do research, losing time for our Ph.D. degree. Our study work is most affected because of the spread of COVID-19 and the stopping of academic activities.”* Some participants described that *“we have to learn online because of the spread of COVID-19. This online learning is quite difficult because we have a hard time understanding what the lecturer is saying. Next, when many students enter the online conference applications, the apps make a noisy sound, interrupting the learning process. Then, the lecturers and the students cannot have effective Q & A sessions because of the time limit in the apps*.” Some participants explain that *“we cannot go to our university, we know there are online classes, but they don’t affect like offline classes. Face-to-face teaching is better than online teaching*.” Under this theme, some participants because of COVID-19; the children stopped studying at school, which created crises for their parents.

Subtheme (2): Family Life and Friendship interruption

The latent theme “family life and friendship”, focused on the influence of the COVID-19 quarantine on family life and friendship, such as relationships and interactions. Participants reported negative features of coronavirus, including the inability to meet family members, husbands, children, and parents, to visit parents in their hometown, to be close to friends, and not to meet friends and live a normal life like before.

In contrast, some cases reported positive social aspects for the COVID-19 quarantine, such as attaching more to family, having more time for family, and re-achieving a good relationship among family members.

Subtheme (3): Job/Work and Business interruption

Under this theoretical theme called “Job/Work and Business”, participants described the idea of losing a job, work, or business either fully or partially. In addition, the participants reflected on their experiences as workers or people in business. In this category, many participants reported that the COVID-19 quarantine affected their professional lives regarding jobs, work, and business. The negative aspects of this latent category can be seen from the participants’ responses, which were centered around the following ideas: “*Stopping work life, unable to go to work or apply or search for jobs, working from home, work is delayed, every task must be done from home, work has stopped, becoming unemployed, reduced working hours, lost part-time job, businesses are affected badly, the company that I have been working with has gone bankrupt, so my source of income has stopped*.”.

Subtheme (4): Psychopathological Pressure

This theme was labeled “psychopathological pressure.” Under this theme, participants reported that the COVID-19 quarantine affected their lives psychopathologically. The quarantine affected participants, who suffered symptoms of depression, anxiety, and insomnia. Participants in the latent category of psychopathological outcome reported that quarantine made them suffer from several symptoms, including “*serious insomnia and psychological stress, claustrophobia, constant fear, worrying, unreasonable worries, being overwhelmed with anxiety, feeling depressed, loneliness, stomach problems, mental trauma, nightmare, changed my sleeping patterns, feeling like in prison, mentally and physically disturbed, getting angry and stressed for no reason, being less social, lonely and distanced from others, quarantine made some cases nervous when meeting with others, my life boring, less active and less energetic, life no longer productive*”.

Subtheme (5): Idly Staying at Home

The COVID-19 quarantine forced participants to stay at home. Participants reported that staying at home generated negative consequences such as working from home and reducing family income, blocking what will happen in the future, prolonged time that changed participants psychologically and mentally, inability to live normally, and hampered normal daily life. Some cases reported that staying home for long periods was horrible.

Subtheme (6): Movement Restrictions

The COVID-19 quarantine affected participants’ lives by restricting movement and freedoms. Participants reported aspects of restrictions in homogenous ideas such as “*unable to go outside, no freedom, limited movement, unable to go anywhere, restricted movement, and restricted to the home*.”

Subtheme (7): Mixed Issues (Miscellaneous)

The mixed issue theme was coded by participants’ responses that described more than one of the previously mentioned issues, such as “*job and restrictions of movement, psychological outcome and stopping the study*.” This theme included the participants who reported that “*coronavirus quarantine also changes their lifestyles radically, destroyed many things in life, coronavirus quarantine changes routine life 100%*.” The miscellaneous includes the conditions such as failure to gather with family or passing time with family, failure to do social work, physical bulkiness, relationship breakup, failure to join celebrations or festivals, loneliness, Failure to search for jobs or works, family relationship conflict due to passing long time at home, failure to learn new skills. Some respondents mentioned COVID-19 as a blessing to learn new technical and application skills. In brief, this theme included either participants who reported more than the previously mentioned themes or reported that the COVID-19 quarantine changed their lives.

Subtheme (8): Income Loss or No Income

The COVID-19 quarantine affected participants’ lives economically. Participants reported that “*income aspects of coronavirus quarantine in several ideas no income, the family leader is facing some financial consequences, less income of the family, delayed salary, reducing global income*.”

Subtheme (9): Time Management

The COVID-19 quarantine influenced and hampered participants’ time management. Time management effects were expressed by participants as follows: “*feeling disappointed with my schedule, the sleeping schedule is messed up, affected schedule of study, the rhythm changed in time, we cannot do daily activities, we cannot have free time as I do if I am at the hostel, curious about graduation on time, and facing difficulties with financial conditions*.”

Subtheme (10): No Travel Tour

The COVID-19 quarantine influenced and hampered participants’ travel behaviors. Travel behavior effects were expressed by participants as follows: “*cannot travel, following up job interviews and other life activities has been stopped, unable to return to home country, cannot travel to visit family, and failed to travel to my country*.”

### 4.2. Quantitative Analysis Results

In the quantitative section, this study analyzed the effect of GAD, PHQ, and ISI on IES-R. The proposed hypotheses were that GAD, PHQ, and ISI significantly affected IES-R due to COVID-19.

#### 4.2.1. Invariance Measurement

The results in Table 3 indicate no significant differences among country samples based on four constructs. F-values were 1.251, 1.329, 0.934, and 1.056, and *p*-values were 0.106, 0.057, 0.614, and 0.366 for PHQ, GAD, ISI, and IESR, respectively. The effect size of all observed variables was eta square η^2^ = PHQ (0.069), GAD (0.073), ISI (0.053), and IESR (0.059).

#### 4.2.2. Measurement Model

**Figure 1:**
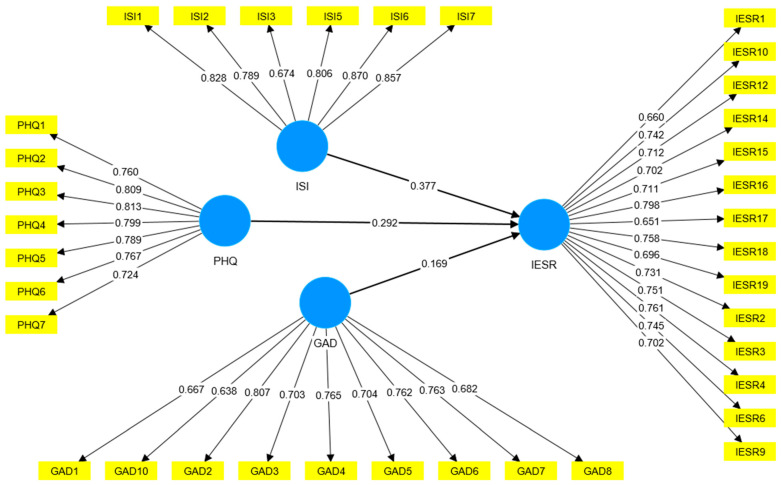
Measurement model.

The findings showed that the model fits with the data and achieved all required values for convergent validity, reliability, discriminant validity, etc., as shown in Table 4 and Table 5, and Figure 1. In line with established guidelines [112,123,124,125], a factor loading threshold of 0.50 was considered acceptable in our study. Factor loadings exceeding this threshold were considered significant indicators of the latent variables, indicating their ability to capture the underlying constructs. Cronbach’s Alpha value was more than 0.70, which reached the optimum level. Composite reliability (CR) was more than 0.70, which is also accepted. The average variance extracted (AVE) was more than 0.50 (Table 4), which was also acceptable. The chosen threshold values for CR and AVE are supported by established guidelines [112,123,124,125]. As for discriminant validity (Table 5), the Fornell and Larcker criterion showed that the square root of AVE is higher than the diagonal value. From the Heterotrait-Monotrait Ratio (HTMT), it was found that all values are less than 0.85, which is permissible. We used a threshold value of 0.85 for HTMT, which is widely accepted in the literature [126,127,128]. Thus, the measurement model indicated moving toward testing the key hypotheses.

#### 4.2.3. Structural Model

**Figure 2:**
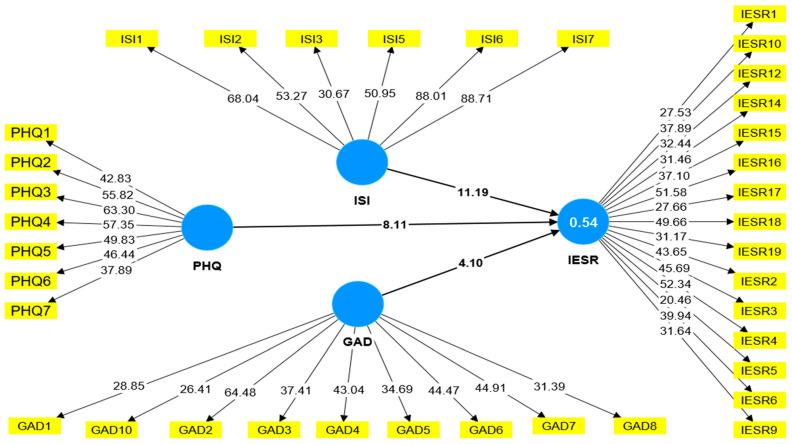
Structural model.

The structural model was evaluated in this phase, including an assessment of potential collinearity issues. We examined all constructs’ Variance Inflation Factor (VIF) values to test for multicollinearity, as presented in Table 6. The results indicated that all VIF values were below the recommended threshold of 3.3, suggesting the absence of significant multicollinearity concerns in our model [108,111,114]. PLS-SEM assumes a 95% confidence interval, with a 5% significance level in 5000 subsamples for bias-corrected bootstrapping to test the hypotheses. The hypotheses are accepted once the coefficient is more than 0.10 with a z-value greater than 1.96 and a *p*-value less than 0.05. The findings showed that all hypotheses were accepted because they met the threshold value. Thus, H1, H2, and H3 were accepted (shown in Table 6 and Figure 2).

## 5. Discussion

The first objective of this study was to delve into the psychological experiences related to (i) anxiety factors about the spread of COVID-19, and (ii) the effects of COVID-19 quarantine on everyday life. The findings revealed four main themes and several subthemes, reflecting the reasons for anxiety due to the spread of COVID-19. These reasons echo findings from prior studies, which identified fears of death, the perception of COVID-19 as a dangerous disease, the absence of a vaccine, the rapid rate of infection, limited healthcare for the heavily infected population, consequences of lockdown and quarantine measures, and the anticipated impact on family and economy as significant sources of anxiety [21,129,130,131,132,133,134,135,136,137,138,139,140,141].

The impact of the COVID-19 quarantine on life was expressed through the effects associated with the quarantine’s main theme, which encompass academic and school life changes, job/work and business operations, family life and friendships, psychopathological outcomes, income, travel, and time management implications. Several studies align with these findings, asserting that COVID-19 quarantine had marked effects on academic life [21,142], work and business [143,144,145,146], family relationships [147,148], psychopathological outcomes [149,150,151,152,153], income [154], travel [144,155,156], and time management [157,158,159]. Quantitatively, the trauma caused by COVID-19 was positively influenced by psychopathological outcomes like depression, anxiety, and insomnia. This concurs with the results of preceding studies [34,35].

Interestingly, some themes overlapped between the reasons for anxiety regarding COVID-19 spread and the effects of the COVID-19 quarantine on life. This overlap reinforces the validity of the two dimensions: the causes for anxiety regarding COVID-19 spread and the impacts of the COVID-19 quarantine on life. However, in the context of qualitative research involving open-ended questions, even with similarities, the responses cannot be consolidated into a single theme. For instance, “staying at home” might be a common response to both questions, but it represents different aspects under different contexts. This underlines the complexity of the hypothetical mixed model and its alignment with the advanced design inconsistencies proposed by Creswell and Creswell [84]. It is noteworthy to remember that such overlapping themes are common in qualitative research [84]. While the pandemic may have ended, future pandemics are always possible. As a result, these findings can provide valuable insights for policymakers and healthcare workers in addressing the long-term mental effects of COVID-19. Furthermore, these results can serve as a guide for dealing with potential future pandemics.

### 5.1. Research Implications, Limitations, and Future Directions

#### 5.1.1. Practical Implications

The findings of this study have important practical implications for various stakeholders, including healthcare providers, policymakers, and community organizations. These implications provide valuable insights into the psychological impact of COVID-19 and inform appropriate strategies to support individuals and communities in the post-pandemic period, especially considering the long-term psychological effects. Healthcare providers can utilize these findings to understand the needs of patients experiencing pandemic trauma and develop targeted intervention strategies for individuals dealing with anxiety, depression, and sleep disorders related to COVID-19. Moreover, policymakers can consider allocating resources to mental health services and implementing strategies to mitigate the anxiety and psychological effects associated with pandemics. Community organizations can use these findings to develop initiatives and services that address the specific needs of people during pandemics, such as providing mental health resources and strengthening social networks.

The study sheds light on the psychological experiences related to anxiety regarding the spread of COVID-19 and the impact of quarantine on daily life. It identifies key factors driving anxiety, including fear of death, the severity of COVID-19, rapid infection rates, and the absence of a vaccine response. Healthcare providers should develop strategies to provide coherent, easy-to-understand, factual information about the pandemic, focusing on preventive measures, treatment protocols, and ongoing vaccine research and development progress to alleviate common anxiety. Future studies can further investigate these experiences and explore additional factors contributing to anxiety during the pandemic and its long-term effects. Researchers can also conduct quantitative studies to measure the prevalence and severity of anxiety in different populations and assess its long-term effects. The study identifies multiple subthemes that reflect the causes of anxiety related to the spread of COVID-19 and the impacts of quarantine on various aspects of life. Given the evident effects of quarantine on academics, professional life, personal relationships, and mental health outcomes, there is an urgent need for supportive policies. These policies may include provisions for distance learning, flexible working arrangements, and widely accessible mental health services, especially during quarantine. Additionally, the study highlights community organizations’ role in mitigating quarantine’s consequences, such as loss of income, travel restrictions, and disruption of daily routines. Initiatives such as financial aid programs, virtual social events, and online career guidance can be highly beneficial.

Furthermore, the study emphasizes the relationship between negative psychological outcomes, including depression, anxiety, insomnia, and trauma caused by COVID-19. Future research can explore the underlying mechanisms of this relationship and investigate possible interventions to mitigate the psychological impact of the pandemic. Additionally, considering the interaction between the causes of anxiety and the effects of quarantine on life, a comprehensive approach that addresses both dimensions is necessary. Researchers can employ mixed method designs to capture the complexity of these experiences and gain a more nuanced understanding of the psychological effects of COVID-19. These research findings can guide future investigations into the psychological aspects of COVID-19, inform public health interventions, and contribute to developing effective strategies to manage anxiety and improve mental health during times of crisis.

#### 5.1.2. Theoretical Contributions

This study makes several notable theoretical contributions to the existing literature. First, it offers a deeper understanding of the impact of the pandemic and subsequent quarantine on individuals’ psychological well-being and lifestyle, thereby expanding the existing body of knowledge on the psychosocial aspects of pandemics. By examining the psychological ramifications of the pandemic and quarantine, the study provides valuable insights into individuals’ distinct challenges during such crises, illuminating the human experience within these circumstances. Secondly, this study contributes significantly by integrating qualitative and quantitative research methods. By employing both approaches, the study captures numerical measures of psychological impact while delving into the qualitative themes underlying these experiences. This integration enables a more comprehensive interpretation of the phenomena, allowing researchers to gain a deeper understanding of the complex interaction between psychological factors and the broader social context.

#### 5.1.3. Limitations and Future Directions

While the present study offers valuable insights into the psychological experiences and life implications of COVID-19, it is essential to acknowledge the limitations and scope of future research. Firstly, although we included participants from 20 countries, which enhanced geographical breadth, this limited us from testing measurement invariance. So, the lack of testing for latent measurement invariance is one of the limitations. As a result, we could not perform multi-group comparisons in PLS-SEM without the measurement invariance information, and our sample’s heterogeneity could limit the results’ comparability across different countries. While providing a broad view, this diversity might also obscure certain country-specific effects. Secondly, despite a sizable qualitative data set collected from 999 participants, we limited our approach to open-ended questions about COVID-19, excluding other qualitative data collection tools such as focus group discussions and interviews. This approach, while efficient, might have missed deeper individual perspectives and narratives.

It should be noted that while the study included participants from various countries with diverse socio-demographic backgrounds, the questionnaire was conducted only in English. As a result, it is possible that some participants may not have had a sufficient level of English proficiency to complete the survey accurately. Future research may benefit from translating or adapting the survey instruments into different languages to mitigate any potential response bias stemming from language barriers. This could help ensure that all participants can fully and accurately participate in the study, regardless of their language background. Moreover, the current study primarily included participants with high levels of education as compared to those with a high school equivalent education. Therefore, for future studies, a random sampling method could be employed to ensure an equal representation of participants, with each individual having an equal chance of being selected. This would help in generalizing the study results.

Another limitation relates to our choice of the partial least square of the structural equation model for simultaneous analysis of mixed and qualitative data. While this method was adequate for our study, other equally potent tools, such as triangulation or multi-trait multi-methods, were not employed. Additionally, despite many responses to the open questions, the sample size in the context of qualitative research might still be considered limited. We should also note that self-report measures could lead to potential social desirability and recall biases. Future research could incorporate objective measures or corroborate self-report data with other sources to overcome these biases.

This study also offers a cross-sectional snapshot of experiences during a specific time frame in the ongoing COVID-19 pandemic. Future studies could adopt a longitudinal design to track the evolving impacts over time. Moreover, future work could also examine specific subgroups, including individuals with pre-existing mental health conditions, frontline healthcare workers, and those who have suffered personal losses due to the virus. These focused investigations could contribute to a more nuanced understanding of the pandemic’s impact. Finally, although we identified key themes related to the pandemic’s impacts, the scope of this study did not extend to the development or testing of interventions to address these impacts. Future research could use these findings to create targeted interventions and evaluate their efficacy. Although some experiences may contain negative dimensions, positive psychology’s second and third waves suggest that every experience is multifaceted and can also contain positive aspects. Recent research, which has not yet been published, indicates that the COVID-19 pandemic has helped some individuals find meaning and make choices that align with their core values. It would be beneficial for future research to incorporate a theoretical framework, such as second/third-wave positive psychology or existentialism, to provide a deeper understanding of these findings. In conclusion, this study contributes to the growing body of literature underscoring the significant impacts of the COVID-19 pandemic across various life domains. As we continue to grapple with these challenging times, research of this nature remains crucial in guiding evidence-based interventions and policy decisions.

## 6. Conclusions

This study comprehensively analyzes the psychological impacts and lifestyle changes resulting from the COVID-19 quarantine. By identifying and examining key themes in participants’ experiences, we have highlighted the various sources of anxiety and the multifaceted effects of quarantine. Our findings underscore the widespread reach of the pandemic, emphasizing the immediate need for targeted interventions in numerous domains. Key factors contributing to COVID-19-related anxiety, including perceived threats to life, shortage of health care services, fear of infection, and family and economic stressors, were identified. Furthermore, our exploration of quarantine’s impacts on daily life revealed a positive correlation with psychopathological outcomes. However, the severity of COVID-19 varies among countries, and the post-traumatic experiences also differ across nations. Additionally, various situations or circumstances can affect post-traumatic symptoms, and residents of different countries may exhibit significant differences in post-traumatic symptomatology. These findings highlight the critical interaction between qualitative and quantitative data within mixed-method designs, reinforcing a holistic understanding of the psychological experiences and consequences of the COVID-19 pandemic. To address these findings, actionable responses are necessary. Educational institutions and workplaces should initiate campaigns to improve self-coping strategies among individuals. Additionally, readily accessible community-based psychological support should be integrated as an intervention to address and alleviate the wide-ranging challenges posed by COVID-19 effectively. The urgency of these initiatives cannot be overstated, considering the pervasive psychological and life-altering ramifications highlighted in our findings.

## Figures and Tables

**Table 1:** Anxious reasons for the spread of coronavirus.

Main Themes	Sub-Themes	Frequency	Percent
Life threats	Death (increasing number of deaths)	123	12.31%
	Life-threatening disease	32	3.20%
	Highly infectious	269	26.93%
Shortage of support	Shortage of vaccine/treatment	52	5.21%
	Unavailability of enough healthcare	29	2.90%
Economic impact	Lockdown and quarantine	31	3.10%
	Economic shutdown	52	5.21%
	Joblessness	47	4.70%
Family and social life	Anxious about family	19	1.90%
	Imbalance in social life	32	3.20%
	Total	686	68.67%

**Table 2:** Effects of the coronavirus quarantine on life.

Main Theme	Sub-Themes	Frequency	Percent	Cumulative Percent
Effects associated with the quarantine	Academic and schooling life interruption	190	19.0%	19.0%
	Family life and friendship interruption	29	2.9%	21.9%
	Job/work and business interruption	95	9.5%	31.4%
	Mixed issues (miscellaneous)	198	19.8%	51.2%
	Psychopathological pressure	102	10.2%	61.4%
	Movement restrictions	35	3.5%	64.9%
	Idly staying at home	51	5.1%	70.0%
	Income loss or no income	39	3.9%	73.9%
	Time management	12	1.2%	75.1%
	No travel or tour	15	1.5%	76.6%

**Table 3:** Invariance measurement.

		Sum of Squares	df	Mean Square	F	Sig.	Eta Value	Eta Square Value
Mean_PHQ	Between groups	33.155	56	0.592	1.251	0.106	0.263	0.069
	Within groups	445.673	942	0.473				
	Total	478.828	998					
MeanGAD	Between groups	31.891	56	0.569	1.329	0.057	0.271	0.073
	Within groups	403.620	942	0.428				
	Total	435.511	998					
Mean_ISI	Between groups	24.959	56	0.446	0.934	0.614	0.229	0.053
	Within groups	449.646	942	0.477				
	Total	474.606	998					
Mean_IESR	Between groups	32.379	56	0.578	1.056	0.366	0.243	0.059
	Within groups	515.578	942	0.547				
	Total	547.958	998					

**Table 4:** Reliability and convergent validity.

Construct/Items	Factor Loadings	Alpha	CR	AVE	R Square
GAD1	0.667	0.89	0.91	0.52	0.54
GAD2	0.807				
GAD3	0.703				
GAD4	0.765				
GAD5	0.704				
GAD6	0.762				
GAD7	0.763				
GAD8	0.682				
GAD10	0.638				
GAD1	0.667				
IESR1	0.660	0.93	0.94	0.52	
IESR2	0.731				
IESR3	0.751				
IESR4	0.761				
IESR6	0.745				
IESR9	0.702				
IESR10	0.742				
IESR12	0.712				
IESR14	0.702				
IESR15	0.711				
IESR16	0.798				
IESR17	0.651				
IESR18	0.758				
IESR19	0.696				
ISI1	0.828	0.89	0.91	0.65	
ISI2	0.789				
ISI3	0.674				
ISI5	0.806				
ISI6	0.870				
ISI7	0.857				
PHQ1	0.760	0.89	0.92	0.61	
PHQ2	0.809				
PHQ3	0.813				
PHQ4	0.799				
PHQ5	0.789				
PHQ6	0.767				
PHQ7	0.724				

Note: CR = composite reliability; AVE = average variance extracted; GAD = generalized anxiety disorder; IESR = revised impact of event scale; ISI = Insomnia Severity Index; PHQ = patient health questionnaire.

**Table 5:** Discriminant validity.

	Fornell-Larcker Criterion				Heterotrait-Monotrait Ratio (HTMT)			
	GAD	IES-R	ISI	PHQ	GAD	IES-R	ISI	PHQ
GAD	0.723							
IESR	0.643	0.724			0.686			
ISI	0.709	0.663	0.806		0.797	0.698		
PHQ	0.708	0.627	0.570	0.781	0.792	0.675	0.636	

GAD = generalized anxiety disorder; IESR = revised impact of event scale; ISI = Insomnia Severity Index; PHQ = patient health questionnaire.

**Table 6:** Path coefficient and hypothesis decision.

Hypotheses	Path Coefficient	Standard Deviation	T Statistics	*p*-Values	VIF	Lower Limit	Upper Limit	Decision
GAD -> IESR	0.168	0.040	4.100	<0.001	2.057	0.096	0.246	Accepted
ISI -> IESR	0.381	0.032	11.19	<0.001	2.793	0.306	0.437	Accepted
PHQ -> IESR	0.290	0.034	8.11	<0.001	2.002	0.222	0.355	Accepted

GAD = generalized anxiety disorder; IESR = revised impact of event scale; ISI = Insomnia Severity Index; PHQ = patient health questionnaire.

## Data Availability

The dataset supporting this study’s findings is not openly available and will be available from the corresponding author upon reasonable request.

## Supplementary Materials

The following supporting information can be downloaded at: https://www.mdpi.com/article/10.3390/healthcare11172418/s1, Table S1: Socio-demographic characteristics of participants (*n* = 999).
