# Supplementary material for: Psychological Toll of the COVID-19 Pandemic: An In-Depth Exploration of Anxiety, Depression, and Insomnia and the Influence of Quarantine Measures on Daily Life
Source: Healthcare (Basel). 2023 Aug 29;11(17):2418. doi: 10.3390/healthcare11172418 (PMC10487588; doi:10.3390/healthcare11172418)
Supplement: Supplementary file 1 [file healthcare-11-02418-s001.zip › healthcare-2531454-supplementary.pdf]

Table S1. Socio-demographic characteristics of participants (N=999).

Variable	n (Percentage)
**Gender**	
Female	554 (55.5%)
Male	445 (44.5%)
**Age**	
18-25	242 (24.2%)
26-35	403 (40.3%)
36-45	254 (25.4%)
46-75	100 (10%)
**Education level**	
High school equivalent	31 (3.1%)
Bachelor	285 (28.5%)
Diploma	79 (7.9%)
Master	367 (36.7%)
PhD	237 (23.7%)
**Country of origin**	
Malaysia	282 (28.2%)
Yemen	111 (11.1%)
Indonesia	73 (7.3%)
Nigeria	62 (6.2%)
Sri Lanka	65 (6.5%)
Tunisia	43 (4.3%)
Pakistan	27 (2,7%)
Somalia	24 (2.4%)
Syria	22 (2.2%)
Saudi Arabia	22 (2.2%)
Vietnam	23 (2.3%)
Bangladesh	29 (2.9%)
China	23 (2.3%)
India	20 (2.0%)
Iraq	34 (3.4%)
Egypt	22 (2.2%)
Algeria	24 (2.4%)
Guinea	21 (2.1%)
Afghanistan	25 (2.5%)
Others	47 (4.7%)
**Marital Status**	
Married	496 (49.6%)
Single	464 (46.4)
Engaged	27 (2.7%)
Divorced	12 (1.2%)
**Employment Status**	
Students	551 (55.2%)
Educational profession	230 (23%)
Administrative professional	56 (5.6%)
Healthcare workers	53 (5.3)
Others	109 (10.9)
